# Pharmaco-Omics in Psoriasis: Paving the Way towards Personalized Medicine

**DOI:** 10.3390/ijms24087090

**Published:** 2023-04-11

**Authors:** Charalabos Antonatos, Paschalia Asmenoudi, Mariza Panoutsopoulou, Yiannis Vasilopoulos

**Affiliations:** Laboratory of Genetics, Section of Genetics, Cell Biology and Development, Department of Biology, University of Patras, 26504 Patras, Greece; charisantonatos@gmail.com (C.A.);

**Keywords:** psoriasis, pharmacogenomics, pharmacotranscriptomics, precision medicine, pharmaco-omics

## Abstract

The emergence of high-throughput approaches has had a profound impact on personalized medicine, evolving the identification of inheritable variation to trajectory analyses of transient states and paving the way for the unveiling of response biomarkers. The utilization of the multi-layered pharmaco-omics data, including genomics, transcriptomics, proteomics, and relevant biological information, has facilitated the identification of key molecular biomarkers that can predict the response to therapy, thereby optimizing treatment regiments and providing the framework for a tailored treatment plan. Despite the availability of multiple therapeutic options for chronic diseases, the highly heterogeneous clinical response hinders the alleviation of disease signals and exacerbates the annual burden and cost of hospitalization and drug regimens. This review aimed to examine the current state of the pharmaco-omic approaches performed in psoriasis, a common inflammatory disease of the skin. We sought to identify central studies that investigate the inter-individual variability and explore the underlying molecular mechanisms of drug response progression via biological profiling in psoriatic patients administered with the extended therapeutic armamentarium of psoriasis, incorporating conventional therapies, small molecules, as well as biological drugs that inhibit central pathogenic cytokines involved in the disease pathogenesis.

## 1. Introduction

While the contribution of genetic variation in clinical remission has been observed throughout multiple case studies depicting the association between polymorphisms and adverse drug effects, pharmacogenetics arose as a scientific field in the late 1950s [1,2]. Nowadays, the development of abundant therapeutic approaches targeting diverse molecules in various disease types has established pharmacogenetics as an essential domain of personalized medicine since germline variants involved in the drug metabolism pose as an indulging approach to determine a priori the most efficacious administration, even in the oncology field where the accumulated somatic mutations during the tumor progression often lead to increased drug resistance and toxicity [3,4]. Such variants, both germline and somatic, refer to single nucleotide polymorphisms (SNPs) located in both coding and non-coding regions of the genome, altering the protein structure or gene expression as well as copy number variations (CNVs) which introduce significant variation through deletion or duplication events in the population and trait risk.

Inter-individual variability in response to therapy displays a heterogeneous heritability estimate, with genetic variants participating in response to therapy accounting for up to 30% [5,6], while interethnic discrepancies strongly affect the genetic predisposition of drug response in the context of warfarin [7]. Nevertheless, the clinical validity of pharmacogenetic variants in more than 500 therapeutic molecules has been identified by the U.S. Food and Drug Administration (https://www.fda.gov/, accessed on 20 February 2023), while the PharmGKB project provides curated knowledge of the genetic impact on the drug response variation [8]. For instance, the clinical stratification of patients in accordance with the *CYP2D6* phase 1 metabolizing enzyme variants [9] is established via the clarified molecular implication of the enzyme in the drug metabolism [10], accounting in some cases for up to 95% of the total phenotypic variation [6].

Genetic approaches in the pharmacogenetics field have evolved from candidate-gene approaches, focused on the mechanism of action of each drug, to genome-wide approaches via large-scale, genome-wide association studies (GWASs), advancing, therefore, the terminology into pharmacogenomics. GWASs allow the thorough evaluation of polygenic traits, as is the case with the heterogeneous drug response, with multiple loci affecting a single trait through gene–gene interactions and epistasis. However, such interactions are not able to be fully captured through solely genome sequencing; holistic approaches are of paramount importance in order to explain the clinical variability as well as predict the response to therapy. In the post-genomic era, the development of various “omics” technologies has resulted in their application in the area of personalized medicine, uncovering potential biomarkers of drug response and toxicity [11]. Biomarkers that represent the functional responder-non-responder variation include but are not limited to, circulating non-coding RNAs [12], proteome expression [13], as well as metabolomic profiling [14]. Such multi-omic approaches could be of particular utility in chronic inflammatory diseases, where lifelong drug administration significantly increases the adverse drug reactions (ADRs) risk [15], while stratification of the patients according to potential response criteria will shrink the annual public health cost [16]. Here, we reviewed the recent pharmac-omic approaches applied in psoriasis, a chronic skin inflammatory disease of steadily increasing prevalence [17].

## 2. Pathophysiology of Psoriasis

The abnormal differentiation and proliferation of keratinocytes occurring in psoriasis lie in the contribution of numerous genetic and environmental factors, with the former explaining more than 70% of the total psoriasis susceptibility [18]. Current knowledge, as derived from GWASs [19] and omic approaches [20], has revealed the intricate interactions between skin and immune cells that occur during both the initiation and the maintenance phases of psoriasis.

**Figure 1:**
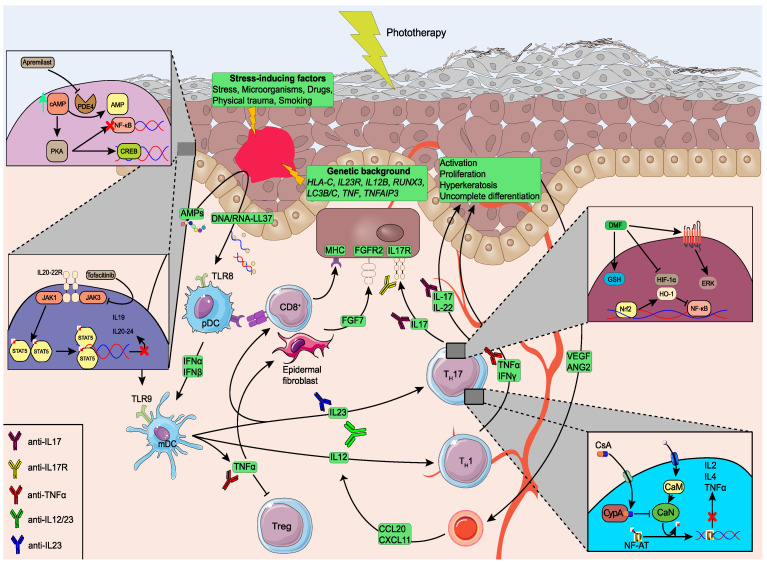
Pathophysiology of psoriasis and major therapeutic approaches.

These interactions are triggered by numerous environmental factors, including trauma through the Koebner phenomenon [21], drug usage [22], and infectious diseases [23]. Such triggering factors pose increased stress over the keratinocytes, leading to the downstream secretion of antimicrobial peptides (AMPs) such as LL37. These AMPs form complexes with self-DNA/RNA, promoting the activation of the Toll-like Receptor 9 (TLR9) signaling pathway in the infiltrating plasmacytoid dendritic cells (pDCs) [24]. Consequently, increased expression of type 1 interferons (IFNα, IFNβ) from the pDCs stimulate the myeloid dendritic cells (mDCs), complementary with the TLR8 signaling pathway incited by the LL37/self-nucleotide complexes [25]. The pivotal role of the above pathways in the transition from the initiation to the maintenance phase of PsO is depicted from the interaction of mDCs with T cell activation via the production of hub pro-inflammatory cytokines and formation of the pathogenic Tumor Necrosis α (TNF)/Interleukin 23 (IL-23)/T helper (T_H_) 17 axis, targeted by multiple therapeutic approaches [26]. In specific, pleiotropic signaling of TNF is expressed from the stimulation of cutaneous fibroblasts and the secretion of the fibroblast growth factor 7 (FGF7), which binds to the keratinocyte FGF receptor 2 (FGFR2) [27,28], as well as exacerbating the inflammatory milieu by limiting the activity of regulatory T cells [29]. Similarly, both mDC-secreted IL12 and IL23 contribute to the psoriatic progression by activating the IFNγ-producing T_H_1 [30] and T_H_17 [31] cells, respectively. Out of those, differentiated T_H_17 comprises the central pathogenic cell subtype of psoriasis, orchestrating the expression of IL-17A, IL-17F, and IL-22, amongst others [31,32]. Both pro-inflammatory cytokine family members are of paramount importance in the pathogenesis of psoriasis, exhibiting a diverse role through the recruitment of neutrophils and the causal interplay with keratinocytes considering the IL-17 family, while IL-22 promotes the keratinocyte proliferation [33]. The described pathways mainly refer to CD4^+^ subpopulations, which have led to the characterization of psoriasis as a T cell-mediated disease [34]; recent research has, however, highlighted the participation of CD8^+^ T cells in the pathogenesis of the disease, present in the healthy skin in the form of skin resident memory T cells (T_RM_) [35]. Stimulated CD8^+^ T cells display a similar inflammatory profile compared to CD4^+^ cells, further categorized according to their secretome [36,37]. Altogether, the indirect, by TNF, and direct, by IL-17s and IL-22 stimulations of keratinocytes lead to the secretion of pro-inflammatory molecules, including chemokines, such as the chemokine (C-C motif) ligand 20 (CCL20) [38], cytokines such as IL-6 [39] and IL-8 [40], and AMPs fostering the maintenance of the inflammatory circuit (Figure 1).

In view of the multifactorial nature of psoriasis, as well as the abundance of molecules involved in the skin-specific inflammatory aggravation, with a small fraction of the deregulated immune pathways described above, several therapeutic approaches have been developed and successfully applied in the clinical routine as a way to control the abnormal hyperproliferation of keratinocytes and, ultimately, alleviate the inflamed symptoms. Nevertheless, the complexity of the disease is maintained in response to therapy, with a significant proportion of psoriasis patients not exhibiting clinical remission [41] as well as exacerbation of ADRs [42]. Clinical remission of psoriasis patients is usually defined as a reduction of the Psoriasis Area Severity Index (PASI) score greater than 75% (PASI75) [43], while ADRs display a drug-specific profile [42]. Therefore, biological profiling of patients undergoing drug administration has been conducted in numerous therapeutic approaches administered in psoriasis, underlying the variation implicated in the subsidence of the psoriatic manifestation.

## 3. Traditional Therapies

Prior to the development of modern approaches, traditional therapies governed and are still maintained in the therapeutic armamentarium of psoriasis. Such examples refer to calcipotriol, a vitamin D analog, the ultraviolet B phototherapy, as well as the traditional systemic agents that include methotrexate and cyclosporine.

### 3.1. Acitretin

Acitretin is a systemic, oral retinoid that binds to retinoic acid receptors (RARs), displaying a distinct anti-inflammatory profile according to the activated RAR [44]. Possible ADRs include dry skin, hair loss, hyperlipidemia, and hepatotoxicity, while it is contraindicated in pregnancy [45].

Predisposition to acitretin response has been investigated in a few pharmacogenetic studies. Contradictory results have been received regarding the association between the *VEGFA* rs833061 variant and acitretin response with a similar sample size [46,47], while negative results were reported regarding *APOE* [48] and *IL36RN* [49] SNPs, implying the assessment of patient subgroups according to additional disease-associated loci. Indeed, deep sequencing of the *HLA* locus uncovered several loci associated with the treatment response (n = 24), with the HLA-DQA1*:02:01, DQB*:02:02 showing the same association signals in the validation set (n = 76) [50]. Variants mapped to ion transporters were also documented to discriminate acitretin responsiveness of 151 Chinese Han psoriasis patients, while functional experiments showed that the *SLCO1B1* rs4149056 C allele reduced the acitretin uptake, resulting in improved treatment efficacy [51].

Serum lipid profiling of 84 acitretin-treated patients revealed significant alterations of triglycerides (TGs) and high-density lipoprotein cholesterol (HDL-C) pre- and post-treatment, as well as when stratified per the *SFRP4* rs1802073 variant [52]. Similarly, circulating levels of the FABP4 protein were significantly increased in psoriasis patients compared to healthy individuals and further reduced to non-pathogenic levels after acitretin treatment [53].

### 3.2. Cyclosporine

Cyclosporine A (CsA) serves as an ideal therapeutic approach in the T cell-mediated pathogenesis of psoriasis, inhibiting the activation of the Nuclear Factor of Activated T cells and downstream IL-2 secretion (Figure 1) [54]. Nevertheless, long-term administration of CsA is usually limited by ADRs, such as nephrotoxicity and hypertension [55].

The majority of pharmacogenetic studies have focused on the mechanism of action of CsA, with the first work reporting a significant association between the rare *ABCB1* rs1045642 allele and negative CsA response in 84 psoriasis patients [56]. The *ABCB1* gene encodes P-glycoprotein facilitating the drug’s influx–efflux into the cell of interest. These results were recently validated in 168 Russian Psoriasis patients receiving CsA for 3 months, further unveiling two associated loci (rs1128503, rs2032582) mapped at the *ABCB1* gene [57]; haplotype analyses from both studies documented that the T-G-C (rs1045642-rs2032582-rs1128503) haplotype frequency was significantly higher in the non-responders group, implying its utilization as a predictive biomarker. Novel approaches have also been conducted in the pharmacogenetics of CsA. Specifically, Antonatos and colleagues attempted to unravel the genetic predisposition of CsA response via reconstruction of the protein interactions during the drug’s mechanism of action and targeted genotyping, showing the association of two genetic variants (*CALM1* rs12885713, *MALT1* rs2874116) with response to CsA treatment in 176 Greek psoriasis patients [54].

Additional pharmacotranscriptomic approaches in the CsA treatment revealed the molecular reversion of several cytokine levels [58,59] and transforming growth factor (TGF)-β isoforms [60], with the former study further displaying the reduction of *K16*^+^, *CD3*^+^, and *CD25*^+^ cells in the lesional skin [58]. Furthermore, immunohistochemical staining for TNF and ICAM-1 of psoriatic skin was significantly reduced after CsA treatment approaching non-lesional levels [61].

### 3.3. Methotrexate

Methotrexate, a synthetic analog of folic acid, has been widely applied in the clinical routine in psoriasis cases, displaying both anti-inflammatory and immunosuppressive mechanisms of action. Despite the significant ADRs its administration provokes, with the exemplar of hepatoxicity, methotrexate remains a widely applied systemic therapy in the clinical routine [62].

Methotrexate encompasses the largest number of pharmacogenetic studies compared to the rest of the conventional therapies, focusing on disease-associated loci, as is the case with the *ANXA6* rs11960458 SNP [63]. Stratification of individuals according to the *HLA-C* allele status revealed significant associations between *HLA-Cw6^+^* patients and treatment outcomes in arthritis-free psoriasis patients [64,65], while the CsA-associated *ABCB1* rs1045642 SNP was additionally incorporated into significant predictive models [65]. In contrast, Yan et al. failed to validate the pharmacogenetic role of the *HLA-Cw6* allele in 90 Chinese patients, reporting, however, associations between the *LCE3D* rs4112788 and *TNIP1* rs10036748 SNPs and responses to therapy [66]. Genes participating in the methotrexate mechanism of action have been further evaluated as potential pharmacogenetic biomarkers in methotrexate-treated psoriasis patients, unveiling variants mapped to efflux transporter *ABCC1*, *ABCG2* [67] and *ABCC2* genes [68], *TYMS* [69], *MTHFR* and *GNMT* [69,70], as well as genes participating in folate, pyrimidine, and purine metabolism pathways [71]. Since methotrexate represents the gold standard of systemic treatment in psoriasis clinical routine, a genome-wide scan was conducted in 333 psoriasis patients and consequently validated in 108 psoriatic individuals. The rs4713429 variant passed the genome-wide significance threshold, further associated with perturbed *HLA-C* expression through expression quantitative loci analysis [72].

The psoriatic expression signals have been shown to be alleviated through methotrexate treatment in several pharmacotranscriptomic studies, with exemplars of the T_H_17-related CCL20 and IL-22 [73], as well as the TNF-like weak inducer of apoptosis *TNFSF12* (*TWEAK*) mRNA and protein levels [74]. Notably, expression profiles from lesional skin from the first 4 weeks of treatment, incorporating four major pharmacotherapies in psoriasis, including methotrexate, were able to accurately predict the clinical endpoint at 12 weeks, setting the framework for the utilization of machine-learning models and expression signatures in precision medicine [75].

Similarly, circulating pathogenic cytokine levels were found to be reduced after methotrexate treatment [76], as well as lesional chemokine CXCL12 levels [77], contrary to the upregulated IL-4 interleukin [76]. Hypothesis-free proteomic analyses have been additionally employed to identify response biomarkers and elucidate the mechanism of action of methotrexate, confirming the molecular reversion of deregulated proteins after administration [78]. Qiu et al. were the first to perform a comprehensive gut microbiome profiling in methotrexate-treated psoriasis patients, attempting to correlate post-treatment microbiome alterations with the blood metabolome [79]. Remarkably, the authors observed discrete clustering patterns according to the baseline metabolomic profiling, reflecting the response to therapy according to the prespecified PASI criteria, while increased microbial diversity pre-treatment was associated with a poor response.

### 3.4. Phototherapy

Phototherapy of psoriasis encompasses a wide variety of irradiations, with the major approaches including narrowband ultraviolet B (nbUVB) and psoralen ultraviolet A (PUVA) approaches, while the latter is often combined in conjunction with oral and/or topical applications [80]. Phototherapy is postulated to induce the apoptosis of T cells and hyperproliferative keratinocytes in psoriasis, accompanied by an increase in T_H_2-related cytokines [80].

Despite their relatively common usage in the clinical routine, research regarding potential response biomarkers in psoriasis phototherapy is limited. In particular, a pharmacogenetic study conducted in 2005 revealed that carriers of the apoptosis-deficient *P53* allele could explain non-response to the UV-based therapeutic approach [81], while homozygous psoriasis patients for the *VDR* rs731236 C allele showed a reduced remission duration [82]. Gene expression microarray analyses further demonstrated that nbUVB phototherapy induced the suppression of T_H_17, IFN signaling, and NF-κB pathways, confirming the molecular reversion patients undergo during efficacious treatment administration (Figure 1) [83,84], a conclusion further depicted through the restored expression of the micro-RNA (miR)-146a after 3 months of therapy [85]. Similarly, phototherapy induced the reduction of serum IL-22 and IL-17 cytokines, confirming thus its anti-inflammatory mechanism of action [86] (Table 1).

## 4. Small Molecules

Small molecules are modulators of the inflammatory pathways present in psoriasis due to their highly infiltratious profile in the dysregulated skin barrier. Their oral or topical administration, as well as their relatively low cost, establish them as promising therapeutic approaches for the alleviation of inflammatory cutaneous signals. Current progress in the depiction of central pharmacological targets has led to the approval of phosphodiesterase 4 (PDE4) inhibitors and fumaric acid esters (FAEs), while Janus kinase (JAK) and Tyrosine kinase 2 (TYK2) inhibitors have been thoroughly evaluated in clinical trials.

### 4.1. Apremilast

Through the inhibition of PDE4, the anti-PDE4 drug regimens increase cAMP levels and alleviate the inflammatory signals, implying their potential clinical efficacy in chronic inflammatory diseases. A major example of PDE4-blocking drugs is apremilast, an approved oral drug regimen for psoriasis (Figure 1).

Hypothesis-free genome-wide scan in 34 psoriasis patients (14 responders) revealed 72 genetic variants located on four chromosomes at a *P* threshold of 10^−6^ as potential heritable biomarkers for apremilast response. Notably, all autosomal variants were mapped to non-coding genomic regions, while the response to treatment indicated an X-linked coding variation inheritance signal through the association of the *ARSF* rs35084576 SNP with the apremilast clinical outcome [87].

The majority of pharmacotranscriptomic and relevant approaches in the anti-PDE4 regimens have focused on the effects of apremilast treatment on isolated patient cells as well as in vivo studies [88,89] attempting to explain the inhibitory mechanisms and direct effects on the samples under study. Protein levels of major pathogenic cytokines in psoriasis were significantly reduced during apremilast administration from a total of 129 patients, contrary to placebo, additionally revealing IL-17F levels as a putative predictor of PASI reduction [90]. The anti-inflammatory action of apremilast is further depicted in the protein levels of relevant pathogenic molecules of psoriasis. Campanati et al. investigated the impact of PDE4 inhibition in angiogenic and inflammatory processes in nine psoriatic apremilast responders, showing significant suppression of VEGF, iNOS, and IDO molecules on psoriatic mesenchymal stem cells after clinical remission [91]. Investigation of plasma biomarkers in 93 apremilast-treated psoriatic patients associated the circulating levels of IL-17A and KLK-7 proteins with both disease severity and response profile, giving valuable insight into the skin biology during disease maintenance and therapeutic administration through the deregulated expression profile of KLK-7 in both mRNA and protein levels [92]. Furthermore, apremilast successfully reversed levels of metabolic markers in 113 psoriasis patients with comorbidities in the non-pathogenic state, such as diabetes and cardiovascular disorders, setting the framework for personalized approaches and stratification of psoriasis patients in the clinical routine [93].

### 4.2. Fumaric Acid Esters

Fumaric acid esters (FAEs), including dimethyl fumarate (DMF), are oral immunomodulatory drugs for psoriasis treatment with a complex mechanism of action (Figure 1). FAEs are usually administered to patients with moderate to severe psoriasis with an inadequate response or partial toleration to other treatments.

Candidate-gene pharmacogenetic approaches are sparse in the FAE investigation, with a single study focusing on the genotyping of the Glutathione S-transferase T1 gene (*GSTT1*) and parallel assessment of the enzymatic activity of the derived enzyme. Results from Gambichler and colleagues displayed a strong positive correlation (rho = 0.95) between *GSTT1* allelic variants and enzymatic activity in 106 psoriasis patients, displaying, however, an absence of association between GSTT1 phenotype and clinical outcomes [94].

Differential microarray analysis of five DMF-treated psoriasis responders depicted the over-expression of anti-inflammatory pathways after treatment response, showing partial intersection with deregulated pathways in four non-responders derived from the same cohort, with exemplars of glutathione signaling and Nrf2 pathways [95]. At the ex vivo level, DMF suppressed the gene expression of major cytokines involved in psoriasis (Figure 1), including IFN-γ, IL-17, and IL-22 in mononuclear cells of both psoriasis patients and healthy individuals [96], inhibiting thus the activity of T_H_1 and T_H_17 cells at the lesional skin.

Circulating markers have also been investigated in FAE administration. Similarly to apremilast, DMF treatment showed a favorable response to cardiovascular parameters and risk markers, including apolipoprotein B and cholesterol reduction in 32 psoriasis patients, with comparable results to 33 adalimumab-treated individuals [97]. Gambichler and colleagues further investigated alterations of AMP levels during DMF treatment, reporting unexpected results regarding the significant increase of all markers under study (LL37, β-defensin-2, and psoriasin) in resolved psoriatic skin [98].

### 4.3. JAK and TYK2 Inhibitors

The JAK/STAT signaling pathway comprises an extended protein network that facilitates the secretion of an abundance of pro-inflammatory cytokines as well as growth factors and thus poses an ideal therapeutic target for psoriasis [99]. Indeed, JAK and TYK2 inhibitors are a class of medications that block Janus kinases and downstream products, showing an efficacious inhibition of pathogenic signaling pathways, including IL-23 (Figure 1). JAK and TYK2 inhibitors, with exemplars of tofacitinib, baricitinib, and deucravacitinib, respectively, display promising results in reducing psoriasis symptoms and improving skin clearance.

Extensive molecular profiling of 267 psoriasis patients undergoing deucravacitinib revealed that TYK2 inhibition restores the epidermal expression of pathologic hallmarks of psoriasis, such as K16, CD3, and Ki-67^+^ cells after 3 months of treatment [100]. In addition, extended transcriptome reversion was observed after effective deucravacitinib treatment, heavily affecting T_H_17 pathways (Table 2).

Contrary to anti-TYK2, JAK inhibitors are not FDA-approved due to the high incidence of ADRs observed during tofacitinib treatment [101]. Nevertheless, several studies have demonstrated the influence of genetic variability in tofacitinib response [102], transcriptomic alterations induced by anti-JAK regimens [103,104], and circulating levels of cardiovascular proteins [105,106]. Stratification of patients according to such biomarkers for the reduction of ADRs might be able to establish tofacitinib and relevant JAK2 approaches in the therapeutic armamentarium of psoriasis.

**Table 2:** Pharmaco-omic approaches applied in small molecules.

Study, Year	Drug	Method	Sample	Time Sample	Clinical Outcome	Time Point	Sample Size	Main Results
Pharmacogenetics								
Gambichler et al., 2013 [94]	DMF	Genotyping	gDNA	n.p.	PASI75	3 months	106	No evidence for association for GSTT1 alleles.
Pharmacogenomics								
Verbenko et al., 2020 [87]	APR	GWAS	gDNA	n.p.	PASI75	6 months	34	Association of the *ARSF* rs35084576 SNP.
Pharmacotranscriptomics								
Onderdijk et al., 2014 [95]	FAEs	Microarray	Lesional skin	Baseline, 12 weeks	PASI75	3 months	50	Overexpression of anti-inflammatory pathways after FAE treatment.
Tahvili et al., 2015 [96]	DMF	RT-qPCR	Plasma	Baseline	n.p.	n.p.	35	DMF suppressed TH1 and TH17 signaling post-treatment.
Catlett et al., 2022 [100]	DEUC	RNA-seq	Lesional skin	Baseline, 2 weeks, 12 weeks	n.s.	3 months	267	Alleviation of central pathogenic molecules post-treatment.
Additional approaches								
Garcet et al., 2018 [90]	APR	Immunoassay	Plasma	Baseline, 2,4,16,24,32,52 weeks	PASI75	4 months	129	Suppression of circulating psoriasis pathogenic molecules, including IL-17 cytokine family and TNF.
Campanati et al., 2020 [91]	APR	ICC, IHC	Lesional skin	Baseline, 12 weeks	PASI75	3 months	9	Suppressed expression of VEGF, iNOS and IDO in keratinocytes.
Medvedeva et al., 2020 [92]	APR	Protein and RNA profiling	Plasma	Baseline	PASI75	4 months	93	Suppression of IL-17A and KLK-7 levels post-treatment.
Mazzilli et al., 2020 [93]	APR	Metabolic profiling	Plasma	Baseline, 24 weeks, 52 weeks	n.s.	12 months	113	APR reversed metabolic markers.
Holzer et al., 2020 [97]	FAEs	Luminex Assay	Plasma	Baseline, 3 months, 6 months	PASI75	3 months	32	FAEs reduce apolipoprotein B and cholesterol.
Gambichler et al., 2012 [98]	DMF, MMF	ELISA	Plasma	Baseline, 3 months	n.s.	3 months	32	AMP levels increased post-treatment.

Abbreviations: DMF, dimethyl fumarate; APR, apremilast; n.p., non-pertinent; N.S., not stated; DEUC, deucravacitinib; RNA-seq, RNA-sequencing; FAEs, fumaric acid esters; ICC, immunocytochemistry; IHC, immunohistochemistry; AMP, antimicrobial peptide.

## 5. Biological Agents

In contrast to the conventional drug approaches described above, biological agents developed throughout recent years have revolutionized the alleviation of psoriatic inflammatory signals and are considered at the forefront of the therapeutic armamentarium of moderate to severe psoriasis. The targeted mechanism of action of such monoclonal antibodies (mAbs), as well as chimeric proteins, allows the inhibition of central cytokines that interconnect the numerous psoriasis-related pathways, such as TNF, IL-17, IL-12, and IL23 (Figure 1), without nevertheless the absence of unresponsive patients which is estimated at 30–50% [107].

### 5.1. Anti-TNF Agents

The first generation of biological drugs in psoriasis consists of the anti-TNF agents (TNF inhibitors; TNFi), incorporating four mAbs (Infliximab, Adalimumab, Certolizumab Pegol, Golimumab) and a single chimeric protein (Etanercept). Despite their long-lasting efficacy in the treatment of multiple TNF-driven autoimmune diseases, the remission heterogeneity and paradoxical psoriasiform reactions prompted the investigation of putative biomarkers of response to therapy.

Candidate-gene approaches have governed the pharmacogenetic studies conducted in the TNFi therapy in patients with psoriasis in search of polymorphisms mapped in *TNF* and susceptibility-related genes. In general, the first study that explored the interindividual variability in responders to anti-TNF therapy was performed in 2012 [108], unveiling the association of the *TNF* rs1799724 and *TNFRSF1B* rs1061622 variants with the positive response to therapy from a total of 80 patients. Multiple studies have henceforth evaluated SNPs mapped in the above genes [109,110], with a pharmacogenetic meta-analysis on European patients confirming the association of common *TNF* (rs361525, *p* = 0.003; rs1800629, *p* = 0.004; rs1799724, *p* = 0.003) and *TNFRSF1B* (rs1061622, *p* = 0.001) alleles and response to anti-TNF therapy in psoriasis [111], while ambiguous results were derived regarding the *FCGR3A* rs396991 SNP (*I*^2^ = 78.9%) [112], a gene responsible for the removal of circulating antigen–antibody complexes. Psoriasis risk variants have also been evaluated in the spectrum of anti-TNF therapy, with a major example of the *HLA-Cw6* allele, an associated susceptibility locus of psoriasis. In spite of its established role in the pathogenesis of the disease, the *HLA-C* locus presents a significant heterogeneity as a pharmacogenetic biomarker of TNFi therapy, failing to be validated in independent cohorts [113,114,115,116,117]. Additional loci that have been extensively studied in the spectrum of anti-TNF therapeutic approaches consist of the TNF-induced protein *TNFAIP3* [114,118,119], the *IL-12B* gene [114,120] and NF-κB-related genes, such as *MIR146A* [121], *NFKBIA*, *TNFR1B* [114], *TLR2* [122] and *NFKBIZ* [113].

Despite their efficacy in the identification of robust genetic risk variants, candidate-gene studies are progressively replaced by hypothesis-free approaches that incorporate genome-wide scans; pharmacogenomic studies have also been performed in psoriasis regarding TNFi approaches, conducted in both Asian [123] and European [124] populations. Genome-wide scans conducted by Ovejero-Benito et al. [124] and Nishikawa et al. [123] included 243 European individuals and 65 Asian psoriatic patients, respectively, receiving 3 months of various anti-TNF therapies, failing to uncover any significant association according to the established GWAS significance thresholds. Both studies, though, implied that treatment-related biomarkers should not be explored in psoriasis-related genes as is the case with *NPFFR2* [124] and *SPEN* [123] genes, but rather than on the mechanism of action of each drug which is yet to be adequately characterized. This hypothesis was further enhanced by the Ren et al. GWA study [125], incorporating two Chinese cohorts who received an etanercept biosimilar for 3 and 6 months. Meta-analysis of both cohorts, comprising a total of 177 patients, characterized the mechanism of action of TNFi therapies despite being unable to reach the significance GWA threshold. In specific, out of the seven loci that displayed an association trend, the rs13166823 SNP was mapped in a cytokine-related locus cluster that has been previously associated with both psoriasis and Crohn’s disease [125]. Nevertheless, two variants mapped in the *CDH12* gene, which was consistently suggested in the previous GWASs, reached nominal significance in the Ren et al. study, giving rise to the need for genome-wide scans with larger sample sizes.

Regarding the transcriptomic landscape of TNFi-treated psoriasis patients, a microarray meta-analysis of lesional skin biopsies showed that the phenotypic reversion observed after successful clinical remission is in concordance with the molecular changes induced by each drug due to the significant association of down-regulated genes with mitosis and keratinocyte-related pathways [126]. Reversion of gene expression to non-lesional levels has also been observed in candidate-gene approaches, including miRNA-146a, miRNA-146b [127], miRNA-125a [128] in plasma samples and members of the Notch signaling pathway in skin biopsies [129] of patients treated with etanercept, skin expression levels of several regulatory elements [130], T_H_17-related cytokines [131,132], and *S100A7* [133] in adalimumab-treated patients and cutaneous *CCL22* [134], *TLR*2 and *TLR9* [135]. However, Suárez-Fariñas et al. postulated that resolved psoriatic lesions do not accommodate a complete molecular reversion of the inflammatory signals, with consistently deregulated genes such as *MMP9*, *CXCR4,* and *IRF1* displaying a less than 75% improvement after TNFi therapy [136]. Whole transcriptome sequencing techniques have also been utilized in the research approaches toward TNFi response prediction. Total RNA sequencing from 18 patients receiving adalimumab enhanced the molecular reversion hypothesis along with the clinical remission, with the most significant pathways referring to the mitotic cycle and keratinocyte differentiation [137]. Co-expression analyses, in addition, unveiled several long non-coding RNA (lncRNA) molecules, not identified at a single-gene analysis level, that interplay in correlated modules significantly enriched for T cell activation (rho = 0.71) and translational regulation (rho = 0.66) [137]. Foulkes et al. examined the transcriptomic landscape of 10 psoriasis patients receiving etanercept in whole blood as well as lesional skin biopsies [138]. Despite the inadequate sample size for the discovery of differentially expressed genes, upstream regulator analysis uncovered a pan-tissue TNF signaling signature that was progressively diminished throughout the treatment administration, while the incorporation of serum proteome profiling depicted a similar pathway pattern, thus enhancing system biology approaches. A larger number of psoriasis patients receiving etanercept were also profiled in a study conducted by Tsoi et al. [139], reaching a total of 42 baseline psoriasis lesional and non-lesional skin biopsies. Notably, baseline expressions showed significant differences only in non-lesional psoriatic skin, with two exceptional examples of the up-regulated *USP18* and down-regulated *KRT2* genes being correlated with the PASI improvement.

Recent evidence also suggests the incorporation of proteomic profiling as a supplementary tool for prediction to therapy, as depicted in the multi-omic analysis conducted by Foulkes et al. through random forest models [138]. Blood proteome profiling of inflammation and cardiovascular-related proteins in 128 patients treated with etanercept displayed a discriminatory ability of 0.7, implying the utilization of skin-related biomarkers due to their superior performance [140]. Additional omic approaches regarding microbiome [141] and epigenomic [142,143,144] profiling induced by the administration of anti-TNF therapies have been recently employed in order to shed light on the mechanism of action of the above drugs, parallelly unveiling putative biomarkers and novel therapeutic targets (Table 3).

### 5.2. Anti-IL23 Agents

The anti-TNF drugs described above paved the way for the development and application of novel mAbs that inhibit the IL23/T_H_17 axis with a cardinal role in the pathogenesis of psoriasis (Figure 1). Ustekinumab, the first representer of the anti-IL23 therapeutic armamentarium in psoriasis, targets the shared p40 shared subunit of both IL12 and IL23, suppressing the inflammatory cascade of T_H_1 and T_H_17 cells, respectively. Additional anti-IL23 drugs have been developed and established in the clinical routine, including guselkumab, tildrakizumab as well as risankizumab, blocking the IL23R receptor.

Pharmacogenetic studies in ustekinumab have also focused on disease-associated loci. Contrary to the TNF inhibitors, however, the *HLA-Cw6* allele has been consistently associated with the response to ustekinumab therapy. A putative explanation for these differences between ustekinumab and TNFi approaches lies in the pathogenic role of IL23 in the polarization of T cytotoxic (Tc) 17 cells that secrete IL17 in the cutaneous inflammation [145], stimulated by the presence of autoantigens that are recognized by the *HLA-C* locus. Indeed, several studies have suggested the HLA-C locus as a pharmacogenetic biomarker, as depicted in a meta-analysis performed by van Vugt et al. [146]. Eight studies comprising 1048 patients displayed a significant favor of *HLA-Cw6* positive patients and response after both 3 (Risk difference (RD), 95%CI = 0.2, 0.11–0.3) and 6 (RD, 95%CI = 0.24, 0.14–0.35) months of ustekinumab therapy. In addition, high-throughput DNA sequencing experiments on 152 psoriatic patients receiving anti-IL12/IL23 therapy for up to two years, assessing the pharmacogenetic role of selected loci, confirmed the role of the *HLA-C* locus in response to therapy [147]. Four, upstream of *HLA-C*, SNPs were associated with a high-response subgroup of patients (achieved PASI90 after 2 years of treatment), with the presence of two variants (rs12189871, rs4406273) combined with the absence of rs9348862 and rs9368670 being able to accurately predict the clinical remission after ustekinumab therapy. Additional associations unveiled by Morelli et al. referred to psoriasis risk loci involving the presence of *PSORS1C3* (rs1265181), *MICA* (rs2523497), and *TNF* (rs1800610) allelic variants, as well as SNPs mapped in the *CDSN* (rs1042127, rs4713436) and the late cornified envelope (LCE) complex (rs12030223, rs6701730) genes. Further association studies regarding the ustekinumab spectrum have successfully associated putative SNPs with the treatment outcome, including a large multicentric Danish cohort identifying *IL1B*, *TIRAP,* and *TLR5* genetic variants (n = 134) [122], *IL12B* SNPs (rs6887695, rs3212227 [148], rs3213094 [149]), and two variants in the *ERAP1* (rs151823, rs26653) gene [150]. Notwithstanding the abundance of pharmacogenetic associations, a single pharmacogenomic approach was not able to validate the above finding in a cohort consisting of 439 European patients treated with ustekinumab for 40 weeks [151]. In specific, Connell et al. identified the absence of the rs35569429 G deletion minor allele (Del-) as a potential pharmacogenetic biomarker (*p* = 2.42 × 10^−9^). Stratification of ustekinumab-treated patients with the independent (*p* = 0.729) *HLA-Cw6* allele enhanced the predictive ability of the rs35569429 variant accordingly since a significantly higher proportion of patients (84.4%) with a Del-/HLA-Cw6 combined genotype achieved the PASI75 endpoint in contrast to the rest subgroups [151] (Table 4).

The transcriptomic landscape induced by the administration of anti-IL23 has been additionally explored in patients with psoriasis. Baseline RNA expression of the p40 subunit, *IL20,* and *IL21* genes was significantly overexpressed in 10 responders to ustekinumab response from a total of 15 psoriatic individuals [152], lacking though validation in an external cohort [153]. Furthermore, studies evaluating the clinical safety of ustekinumab demonstrated a relatively unchanged antimicrobial response accompanied by the molecular reversion of psoriasis-related genes, such as *GATA3*, *DEFB3*, *S100A7,* and *STAT3* [154]. Whole transcriptome profiling approaches have also been incorporated into the pharmacotranscriptomic approaches of ustekinumab treatment. Skin biopsies of 23 ustekinumab-treated patients for 12 weeks were evaluated for putative expression changes via microarray technology; ustekinumab displayed a 97% overall improvement of the perturbed expression profile, as depicted from the suppressed mRNA levels of pathogenic cytokines, including IL23 subunits and IL17A- and IL17-modulated transcripts [155]. Brodmerkel et al. additionally showed significantly higher efficacy of ustekinumab- versus etanercept-treated psoriatic patients, as measured by the molecular reversion of both disease-associated genes and pathways (*p* < 0.05), while their data proposed a stronger modulation of TNF-induced transcripts by ustekinumab. Another head-to-head comparison of ustekinumab with an approved anti-IL23 mAb, risankizumab, provided similar results for both therapeutic approaches [156]. However, risankuzmab induced an earlier molecular reversion in contrast to ustekinumab, parallelly modulating a larger number of transcripts associated with keratinocytes and monocytes [156]. Despite the absence of pharmacogenetic approaches in guselkumab treatment, a microarray analysis of skin tissue biopsies from 24 patients demonstrated a significantly reduced expression of T_H_17-related molecular biomarkers such as S100A7 and CXCL1 after 12 weeks of therapy [157]. Lu et al. explored the differential expression between 37 guselkumab-treated patients for 48 weeks, stratifying the comprised cohort according to the PASI75 improvement score at 4 weeks of therapy [158]. Fast responders displayed a significant increase in natural killer (NK) and low-density neutrophil cell abundance, while up-regulated genes were associated with neutrophil chemotaxis.

Novel approaches regarding guselkumab have also been conducted in the context of drug–protein interactions mediated by the disease-specific profile of psoriasis. Zhu et al. evaluated the pharmacokinetics (PKs) of the anti-IL23p19 drug, exploring its potential interaction with the CYP450 isoenzyme [159]. Sixteen psoriatic patients receiving a CYP probe cocktail along with guselkumab reported mild ADRs, while physical parameters such as vital signs and electrocardiogram did not display a clinically significant difference, highlighting the absence of PK interactions between guselkumab and drugs metabolized by CYP450 enzymes. Additional studies have evaluated the skin microbiome through 16S rRNA sequencing, providing insight into the progressing microbial heterogeneity during ustekinumab administration between lesional and non-lesional skin and recurring psoriasis [160], as well as the lipid composition of blood-derived extracellular vesicles displaying a reversive trend induced by ustekinumab in contrast to other therapeutic approaches [161] (Table 4).

### 5.3. Anti-IL17 Agents

IL17 has been described as a central effector in the pathogenesis of psoriasis, displaying its mechanism of action through keratinocyte stimulation into a hyperproliferative activity and abnormal differentiation (Figure 1). Neutralization of the IL23/T_H_17 forms the third generation of mAbs established in the clinical routine of moderate-to-sever psoriasis cases, consisting of secukinumab, ixekizumab, and brodalumab, with the latter inhibiting the IL-17R receptor (Figure 1).

Pharmacogenetic studies in anti-IL17 therapy have, thus far, investigated the role of the *HLA-Cw6* allele in the therapeutic response. A retrospective study conducted by Anzengruber et al. depicted a non-significant association between the *HLA-C* allelic variation and response to secukinumab; nevertheless, this result came from a total of 18 patients, suggesting the incorporation of a larger number of psoriatic individuals receiving anti-IL17 therapy [162]. This conclusion was additionally confirmed from a larger clinical trial including 185 Cw6-positive and 246 Cw6-negative patients receiving secukinumab for 24 weeks. According to the primary endpoint achieving PASI90 at 16 weeks, no significant associations were found between Cw6-positive and Cw6-negative patients (OR, 95%CI = 0.753, 0.44–1.28), displaying the relatively high clinical efficacy of secukinumab treatment since about 80% of the total patients reached the clinical endpoint. Accordingly, PASI75 was achieved in more than 90% of both groups with the absence of statistical differences in adverse events occurring (*p* = 0.2955) [163]. The extended clinical endpoint, reaching up to PASI75 until week 72, did not show any statistical difference [164], implying the investigation for other loci that might be implicated in the anti-IL17 response. Henceforth, van Vugt et al. performed a pharmacogenetic analysis on a European multicentric cohort of 134 psoriatic patients receiving secukinumab or ixekizumab on the *IL17A* gene [165]. Surprisingly, none of the five analyzed genetic variants mapped in the untranslated regions of *IL17A* were discovered, generating an inconclusive result regarding the genomic variance that influences clinical heterogeneity in anti-IL17 treatment response. However, a targeted pharmacogenomic study consisting of a 417 SNPs panel in 69 European patients unveiled several genetic biomarkers; polymorphisms upstream of the *HLA-C* locus, as well as on the *MICB-DR*, *DDX58*, *TYK2*, and *LTA* genes were found to be statistically associated with the response to therapy [166]. In addition, the stratification of patients according to their *HLA-Cw6* allelic status further characterized the genomic profile of an excellent responders group, similar to the ustekinumab GWAS analyzed above [151]. These results provide an early framework for the participation of the *HLA-Cw6* allele in the biological drug response, further enhancing its role in the pathogenesis and onset of the disease, forming a distinct clinical profile [19,166] (Table 5).

Although no hypothesis-free genomic approach has been conducted, several studies have explored the transcriptomic profile of patients administered with anti-IL17 therapeutic approaches. Krueger et al. performed a gene expression analysis through an Affymetrix microarray platform on skin biopsies derived from 46 patients treated with ixekizumab, aiming to characterize the transcriptomic landscape of patients during ixekizumab administration and its effects on the expression of pathogenic cytokines and keratinocyte-related transcripts [167]. As expected, the majority of the perturbed psoriatic transcripts were normalized after 6 weeks of treatment in a dosage-dependent pattern, associated with both the hyperproliferative activity of keratinocytes and the central cytokines that orchestrate the polarization of CD4^+^ cells. Notably, ixekizumab is further capable of reducing the peripheral IL-17A levels associated with major comorbidities, including cardiovascular diseases and atherosclerosis [168]. Bertelsen et al., on the other hand, were the first to explore the transcriptomic perturbations mediated by secukinumab from lesional biopsies of 18 patients, observing not only the molecular reversion present in all the therapeutic approaches established in the clinical routine but also the rapid attenuation of the NF-κB signature expression after 4 days of secukinumab treatment [169]. Evaluation of the secukinumab treatment effects was further investigated in a cell-specific manner through whole-transcriptome sequencing, depicting a significant reduction of the IL-17 signaling mRNA expression in all the T cell subsets present in the inflamed biopsies of 15 individuals compared to 13 controls as well as the persistence of a small subset of genes including *OAS1*, *OAS2*, and *OAS3*, implying an IL17-unrelated transcriptional regulation [170]. In addition, deconvolution analysis from Liu et al. contrasted with the results of the flow cytometry analysis regarding the T regulatory (T_reg_) sub-populations, suggesting increased immunosuppressive activity in the absence of an increased cellular abundance [170]. Seeler and Moldovan et al. managed to disentangle the transcriptional activity of regulatory elements during secukinumab therapy which showed a rapid molecular reversion upon 4 days of treatment [171]. In specific, the expression of several circular RNAs (circRNAs), including ciRS-7, circPTPRA, and circSCMH1, was negatively correlated with the PASI improvement due to their overexpression across the treatment course, while miRNAs such as miR-223-3p, miR-4454, and miR-15α-5p displayed a positive correlation during 84 days of secukinumab treatment. Pharmacotranscriptomic studies have also explored the brodalumab-mediated expression profile, expressing its immunomodulatory activity through the IL-17R blockade. Russel et al. identified a complete molecular response of individuals receiving higher brodalumab exposure, including both IL-17-related and keratinocyte hyperproliferation-related transcripts [172], with similar results derived from Tomalin et al. who examined a total of 116 patients through microarray profiling and immunochemistry of lesional biopsies [173].

Serum levels of various biomarkers have been incorporated into the understanding of anti-IL17 therapeutic approaches. Thirty-five psoriatic patients receiving ixekizumab (n = 13) and secukinumab (n = 22) displayed a significant improvement in the lipid and inflammatory parameters after 6 months of therapy, nevertheless with the absence of additional improvements in their body compositions parameters, including body mass index (BMI) and body fat percentage [174]. Similar results were derived from Cao et al., where 31 metabolites were significantly deregulated after ixekizumab therapy. In specific, lysophosphatidylcholines (LPCs) and glycerophosphocholine (GPC) levels were drastically ameliorated, while the up-regulation of dicarboxylic acids (Das) suggested a generalized anti-inflammatory action of ixekizumab [175]. A head-to-head comparison of secukinumab and adalimumab in the suppression of uric acid levels unveiled the efficacy of the former in psoriatic patients suffering from hyperuricemia, suggesting its clinical administration in patients with the above comorbidity [176]. Secukinumab has also been compared to ustekinumab under the prism of the gut microbiome, highlighting a distinct mechanism of action between both drugs as depicted by the microbiome shifts [177]. Moreover, Yeh et al. explored the baseline gut microbial composition of secukinumab responders, identifying *Citrobacter*, *Staphylococcus*, and *Hafnia/Obesumbacterium* taxa as putative biomarkers of response to therapy [177] (Table 5).

## 6. Discussion

The major goal of pharmaco-omics refers to the unveiling of molecular biomarkers as efficient predictors of an adequate response profile, parallelly decreasing the relatively high cost of pharmacotherapies. Such approaches aim to accurately describe the inter-individual variability underlying drug administration and clinical remission, fostering thus the development of precision medicine guidelines. Pharmaco-omics in psoriasis have, thus far, covered a significant part of putative biomarkers, showing modest effects nevertheless when applied in real-world practice. Candidate-factor approaches have failed to report validated associations and pinpoint robust molecular biomarkers of clinical remission, stimulating the establishment of hypothesis-free studies. To form ample clinical guidelines for the direct improvement of patient outcomes and quality of life through drug administration, future research should focus on the incorporation of larger sample sizes in such hypothesis-free approaches to validate findings and guide clinical practice, as well as incorporate exposure risk factors into predictive models. For example, the stratification of patients according to modifiable risk factors with an associated implication in the drug response (e.g., BMI) could potentially reveal molecular discrepancies between disease subgroups and thus imply different therapeutic approaches. Similarly, the assessment of relevant comorbidities during the psoriatic inflammation and the derived biological profile might enhance precision approaches, with the exemplar of mental disorders and the overall emotional state of psoriasis patients that aggravate the existing inflammation [178,179]. Holistic approaches through the integration of multi-omic analyses are additionally of paramount importance in the complete understanding of the disease course during treatment administration to identify potential disease- and drug-related biomarkers [75,79,138,140,160]. In addition, the recent establishment of drug switch in the clinical routine, namely the replacement of prescribed drugs as an alternative therapeutic approach, should be additionally explored to assess the extent of biological reversion and possible exploration for secondary response biomarkers. In this way, results emerging from such approaches shall facilitate the identification of subgroup biomarkers and personalized approaches, lower the risk of ADRs, and thus advance the expanding field of precision medicine.

## Figures and Tables

**Table 1:** Pharmaco-omic approaches applied in conventional therapies.

Study, Year	Drug	Method	Sample	Time Sample	Clinical Outcome	Time Point	Sample Size	Main Results
Pharmacogenetics								
Young et al., 2006 [46]	Acitretin	Genotyping	gDNA	n.p.	PASI75	3 months	106	Association of *VEGF* rs833061 SNP.
Chen et al., 2018 [47]	Acitretin	Genotyping	gDNA	n.p.	PASI75	2 months	131	No evidence for associatiation for *VEGF* and *EGF* SNPs.
Campalani et al., 2006 [48]	Acitretin	Genotyping	gDNA	n.p.	PASI75	3 months	190	No evidence for association for *APOE* SNPs.
Zhu et al., 2018 [49]	Acitretin	Genotyping	gDNA	n.p.	PASI75	24 months	109	No evidence for association of *IL36RN* SNPs.
Zhou et al., 2022 [50]	Acitretin	Genotyping	gDNA	n.p.	PASI75	2 months	100	Association of *HLA-DQA1* and *DQB1* alleles
Chen et al., 2018 [51]	Acitretin	Genotyping	gDNA	n.p.	PASI75	2 months	151	Association of *SLCO1B1* rs4149056 and *SLC22A1* rs2282143 SNPs.
Zhou et al., 2018 [52]	Acitretin	Genotyping	gDNA	n.p.	PASI75	2 months	84	Association of *SFRP4* rs1802073 with elevated serum lipid levels.
Antonatos et al., 2022 [54]	CsA	Genotyping	gDNA	n.p.	PASI75	3 months	176	Association between *CALM1* rs12885713 and *MALT1* rs2874116 SNPs.
Vasilopoulos et al., 2014 [55]	CsA	Genotyping	gDNA	n.p.	PASI75	3 months	84	Association of *ABCB1* rs1045642 SNP.
Chernov et al., 2022 [56]	CsA	Genotyping	gDNA	n.p.	PASI75	3 months	168	*Association* between *ABCB1* rs1045642, rs2032582 and rs1128503 SNPs.
Fan et al., 2021 [63]	MTX	Genotyping	gDNA	n.p.	PASI75	12 months	310	Association of *ANxA6* rs11960458 SNP.
West et al., 2017 [64]	MTX	Genotyping	gDNA	n.p.	PASI75	12 months	70	Association of the *HLA-Cw6* allele.
Mao et al., 2022 [65]	MTX	Genotyping	gDNA	n.p.	PASI75	2 months	204	Association of the *HLA-Cw6* allele.
Yan et al., 2019 [66]	MTX	Genotyping	gDNA	n.p.	PASI75	3 months	221	Association of *TNIP1 rs10036748 SNP.*
Warren et al., 2008 [67]	MTX	Genotyping	gDNA	n.p.	PASI75	3 months	374	Association of *ABCC1* and *ABCG2* SNPs.
Grželj et al., 2021 [68]	MTX	Genotyping	gDNA	n.p.	PASI75	6 months	117	Association of *ABCC2* rs717620 SNP.
Voron’ko et al., 2022 [69]	MTX	Genotyping	gDNA	n.p.	PASI75	1 month	139	Association of *MTHFR* and *TYMS* SNPs.
Grželj et al., 2021 [70]	MTX	Genotyping	gDNA	n.p.	PASI75	6 months	199	Association of *GNMT* rs10948059 SNP.
Campalani et al., 2007 [71]	MTX	Genotyping	gDNA	n.p.	PASI75	3 months	203	Association of folate, pyrimidine and pourine gene SNPs.
Hairutdinov et al., 2005 [81]	UVB	Genotyping	gDNA	n.p.	PASI75	1 month	110	Association of the apoptosis-deficient *P53* allele.
Ryan et al., 2010 [82]	UVB	Genotyping	gDNA	n.p.	PASI75	4 months	119	Association of *VDR* rs731236 SNP.
Pharmacogenomics								
Zhang et al., 2021 [72]	MTX	GWAS	gDNA	n.p.	PASI75	3 months	441	Association of rs4713429 SNP.
Pharmacotranscriptomics								
Haider et al., 2008 [58]	CsA	Microarray	Lesional Skin	Baseline, 14 days, 1 month, 2 months	PASI75	1 month	11	Down-regulation of pro-inflammatory cytokines during CsA treatment.
Grabarek et al., 2019 [59]	CsA	RT-qPCR	Plasma	3 months	n.s.	3 months	32	IL12/23 signaling pathway is reversed during CsA treatment.
Michalska-Bańkowska et al., 2018 [60]	CsA	RT-qPCR	Plasma	Baseline, 1 month, 3 months	n.s.	3 months	32	Reversion of TGF-β isoforms.
Goldminz et al., 2015 [73]	MTX	Microarray	Lesional Skin	Baseline, 1, 2, 4, 16 weeks	PASI75	4 months	30	MTX administration shows similar reversion profile to ADA.
El-Esawy et al., 2022 [74]	MTX	RT-qPCR	Plasma	Baseline and 3 months	PASI75	3 months	148	Elevated circulating TNFSF12 mRNA and protein after MTX administration.
Correa da Rosa et al., 2016 [75]	MTX	Microarray	Lesional Skin	Baseline, 1 week, 2 weeks, 1 month	PASI75	1 month	141	Expression profile after 2–4 weeks of treatment accurately predicts the clinical outcome at 3 months.
Rácz et al., 2011 [83]	UVB	Microarrays	Lesional Skin	Baseline, after therapy	PASI75	3 months	11	UVB suppresses inflammatory and skin-related pathways.
Hochberg et al., 2007 [84]	UVB	Microarrays	Lesional Skin	Baseline, 1 month	PASI75	1 month	12	UVB induces the *IGFBP7* expression.
Ele-Refaei et al., 2015 [85]	UVB	RT-qPCR	Plasma	3 months	PASI75	3 months	40	UVB reduced miR-146a levels.
Additional approaches								
Baran et al., 2019 [53]	Acitretin	ELISA	Plasma	Baseline and 12 weeks	n.s.	3 months	33	Circulating FABP4 levels were not altered after acitretin treatment.
Esrefoglu et al., 2006 [61]	CsA	Immunochemistry	Lesional Skin	Baseline, 6 months	n.s.	6 months	10	TNF and ICAM-1 are reduced after CsA treatment.
Indhumathi et al., 2017 [76]	MTX	ELISA	Plasma	Baseline, 3 months	PASI75	3 months	189	Reduced circulating IFN-γ, IL-2, IL-12, IL-23 and increased IL-4 post MTX treatment.
Abdelaal et al., 2019 [77]	MTX	Immunochemistry	Skin	Baseline, 1 month	PASI75	1 month	20	MTX administration supressess lesional CXCL12 expression.
Yan et al., 2022 [78]	MTX	iTRAQ	Plasma	2 months	PASI75	2 months	12	MTX administration normalizes inflammatory expression levels.
Qiu et al., 2022 [79]	MTX	Metagenomics sequencing	Plasma, Stool	Baseline and 4 months	PASI75	4 months	15	Metabolic and metagenomic profiling can predict MTX response.
Lo et al., 2010 [86]	UVB	ELISA	Plasma	Baseline and after therapy	PASI75	n.s.	32	Reduced serum IL-17 and IL-22 levels post UVB treatment.

Abbreviations: n.p., non-pertinent; gDNA, genomic DNA; PASI, psoriasis area severity index; SNP, single nucleotide polymorphism; CsA, Cyclosporine; MTX, Methotrexate; UV, phototherapy; GWAS, genome-wide association study; n.s., not stated; iTRAQ, isobaric tag for relative and absolute quantitation.

**Table 3:** Pharmaco-omic approaches applied in anti-TNF agents.

Study, Year	Drug	Method	Sample	Time Sample	Clinical Outcome	Time Point	Sample Size	Main Results
Pharmacogenetics								
Vasilopoulos et al., 2012 [108]	anti-TNF	Genotyping	gDNA	n.p.	PASI75	6 months	80	Association of *TNF* rs1799724 and *TNFRSF1B* rs1061622 SNPs.
Ito et al., 2019 [110]	anti-TNF	Genotyping	gDNA	n.p.	PASI75	12 months	49	No evidence for association between *TNF*, *TNFSRF1B* and *TNFAIP3* SNPs.
Antonatos et al., 2021 [111]	anti-TNF	Meta-analysis	n.p.	n.p.	PASI75	n.p.	n.p.	Association of *TNF* rs361525, rs1800629, rs1799724 and *TNFRSF1B* rs1061622 SNPs.
Coto-Segura et al., 2019 [113]	ADA	Genotyping	gDNA	n.p.	PASI75	23 months	169	Association of *NFKBIZ* rs3217713 and *HLA-Cw6* alleles.
Ovejero-Benito et al., 2018 [114]	anti-TNF	Genotyping	gDNA	n.p.	PASI75	3 months	95	Association of *IVL* rs6661932, IL-12B rs2546890, NFKBIA rs2146523, ZNF816A rs9304742 and SLC9A8 rs645544 variants.
Talamonti et al., 2017 [115]	ADA	Genotyping	gDNA	n.p.	PASI75	36 months	122	No evidence for association for the *HLA-Cw6* allele.
Prieto-Pérez et al., 2016 [117]	anti-TNF	Genotyping	gDNA	n.p.	PASI75	3 months	144	Association of *PGLYRP4-24* rs2916205, *ZNF816A* rs9304742, *CTNNA2* rs11126740, *IL-12B* rs2546890, *MAP3K1* rs96844 and *HLA-C* rs12191877 SNPs.
Ovejero-Benito et al., 2019 [118]	anti-TNF	Genotyping	gDNA	n.p.	PASI75	3 months	20	Association of *TNFAIP3* rs610604 and rs6920220 SNPs.
Tejasvi et al., 2012 [119]	anti-TNF	Genotyping	gDNA	n.p.	PASI50	6 months	433	Association of *TNFAIP3* rs610604 SNP.
Torii et al., 2020 [120]	IFX	Genotyping	gDNA	n.p.	PASI75	12 months	64	Association of *IL-12B* rs2546890 SNP.
Nani et al., 2023 [121]	anti-TNF	Genotyping	gDNA	n.p.	PASI75	6 months	100	Association of *MIR146A* rs2910164 SNP.
Loft et al., 2017 [122]	anti-TNF	Genotyping	gDNA	n.p.	PASI75	6 months	478	Association of *IL-1B* rs1143623 and rs1143627, *LY96* rs11465996, *TLR2* rs11938228 and rs4696480 and *TLR9* rs352139 SNPs.
Pharmacogenomics								
Nishikawa et al., 2016 [123]	anti-TNF	GWAS	gDNA	n.p.	PASI75	6 months	65	No evidence for association for 731.442 SNPs (P < 5 × 10^−8^).
Ovejero-Benito et al., 2020 [124]	anti-TNF	GWAS	gDNA	n.p.	PASI75	3 months	243	No evidence for association for 584.141 SNPs (P < 5 × 10^−8^).
Ren et al., 2022 [125]	ETA	GWAS	gDNA	n.p.	PASI75	6 months	209	No evidence for association for >350.000 SNPs (P < 5 × 10^−8^).
Pharmacotranscriptomics								
Antonatos et al., 2022 [126]	anti-TNF	Meta-analysis	Lesional Skin	n.p.	PASI75	n.p.	n.p.	Keratinocyte proliferation is repressed after TNF inhibition.
Shen et al., 3033 [127]	ETA	RT-qPCR	Plasma	Baseline, 1 month, 3 months, 6 months	PASI75	6 months	80	Mirs 146a and 146b were gradually over-expressed during ETA treatment.
Pei et al., 2019 [128]	ETA	RT-qPCR	Plasma	Baseline, 1 month, 3 months, 6 months	PASI75	6 months	126	Baseline under-expression of mir-125a in responders.
Skarmoutsou et al., 2015 [129]	ETA	RT-qPCR	Lesional Skin	3 months	PASI50	3 months	16	Notch signaling is repressed after TNF inhibition.
Raaby et al., 2015 [130]	ADA	Microarray	Lesional Skin	3 months	PASI75	3 months	10	Mirs 125a, 203, 21 and 31 displayed a transcriptomic reversion after 3 months of ETA treatment.
Balato et al., 2013 [131]	ADA	RT-qPCR	Plasma, Lesional Skin	Baseline, 4 months	PASI75	4 months	20	ADA administration represses TH17-related cytokines.
Luan et al., 2015 [132]	ADA	RT-qPCR	Plasma	Baseline, 3 months	PASI75	3 months	21	ADA administration decreased pathogenic CD4+ cells and associated transcripts.
Sato et al., 2017 [133]	IFX	RT-qPCR	Lesional Skin	3 months	PASI75	3 months	24	S1007A and IL-8 transcripts were negativelly correlated with response to IFX.
Vageli et al., 2015 [134]	anti-TNF	RT-qPCR	Lesional Skin	Baseline, 3 months	PASI75	3 months	17	TLRs 2 and 9 were significantly reduced post treatment.
Kusumoto et al., 2014 [135]	IFX	RT-qPCR	Lesional Skin	3 months	PASI75	3 months	17	CCL22 and related chemokines are overexpressed in IFX responders.
Suárez-Fariñas et al., 2011 [136]	ETA	Microarray	Lesional Skin	3 months	PASI75	3 months	20	Biologic treatment displays an inadequate molecular reversion.
Ahn et al., 2016 [137]	ADA	RNA-seq	Lesional Skin	1 month	PASI75	1 month	18	Keratinocyte proliferation is repressed after TNF inhibition.
Foulkes et al., 2019 [138]	ETA	RNA-seq, Proteomics	Lesional Skin	3 months	PASI75	3 months	10	TNF-induced mRNA changes are the most predictive of the TNF inhibitor response.
Tsoi et al., 2021 [139]	ETA	RNA-seq	Lesional Skin	3 months	PASI75	3 months	42	Non-lesional USP18 and KRT2 mRNA levels were correlated with PASI improvement.
Additional approaches								
Tomalin LE, Kim J et al., 2019 [140]	ETA	Proximity Extension Arrays	Plasma	Baseline, 1 month	PASI75	3 months	128	Plasma proteome displays a significant, nevertheless inferior to skin proteome predictive accuracy.
Zhao et al., 2022 [141]	ADA	16S rRNA-seq	Stool	Baseline, 3 month	PASI75	3 months	13	Intestinal microbiome was not significantly affected by treatment response.
Nwanaji-Enwerem et al., 2021 [142]	anti-TNF	Methylation	Plasma	Baseline	PASI75	3 months	70	Partial responders displayed a higher Skin-Blood DNA methylation age.
Ovejero-Benito et al., 2017 [143]	anti-TNF	Methylation	Plasma	Baseline	PASI75	3 months	70	No significant methylation changes were observed.
Roberson et al., 2012 [144]	anti-TNF	Pyrosequencing	Lesional Skin	1 month	PASI75	1 month	5	TNF inhibition partially restores perturbed CpG methylation status.

Abbreviations: n.p., non-pertinent; gDNA, genomic DNA; PASI, psoriasis area severity index; SNP, single nucleotide polymorphism; ADA, adalimumab; IFX, infliximab; GWAS, genome-wide association study; ETA, etanercept; RNA-seq, RNA sequencing.

**Table 4:** Pharmaco-omic approaches applied in anti-IL23 agents.

Study, Year	Drug	Method	Sample	Time Sample	Clinical Outcome	Time Point	Sample Size	Main Results
Pharmacogenetics								
van Vugt et al., 2019 [146]	UST	Meta-analysis	n.p.	n.p.	PASI75	n.p.	n.p.	*HLA-Cw6* positive patients showed a favored response to UST therapy.
Morelli et al., 2022 [147]	UST	Genotyping	gDNA	n.p.	PASI75	4 months	152	Association of *HLA-C* variants, *PSORS1C3* rs1265181, *MICA* rs2523497, *TNF* rs1800610, *CDSN* rs1042127 and rs4713436 and *LCE3A-B* rs12030223 and rs6701730 SNPs.
Loft et al., 2017 [122]	UST	Genotyping	gDNA	n.p.	PASI75	3 months	134	Association of *IL1B* rs1143623 and rs1143627, *TIRAP* rs8177374 and *TLR5* rs5744174 SNPs.
Galluzo et al., 2016 [148]	UST	Genotyping	gDNA	n.p.	PASI75	12 months	64	Association of the *HLA-Cw6* allele, and *IL12B* rs6887695 and rs3212227 SNPs.
van den Reek et al., 2016 [149]	UST	Genotyping	gDNA	n.p.	PASI75	3 months	66	Association of *IL12B* rs3213094 SNP.
Masouri et al., 2016 [150]	UST	Genotyping	gDNA	n.p.	PASI75	6 months	22	Association of *ERAP1* rs121823 and rs26653 SNPs.
Pharmacogenomics								
Connet et al., 2021 [151]	UST	Genotyping	gDNA	n.p.	PASI75	12 months	439	Association of *WDR1* rs35569429 SNP.
Pharmacotranscriptomics								
Gedebjerg et al., 2013 [152]	UST	RT-qPCR	Lesional skin	Baseline	PASI75	4 months	15	Baseline over-expression of the p40 subunit in responders.
Zhou et al., 2020 [153]	UST	RT-qPCR	Plasma	Baseline, 3 months, 6 months	PASI75	3 months	24	Increased p40 mRNA throughout the administration of UST.
Baerveldt et al., 2013 [154]	UST	RT-qPCR	Lesional skin	Baseline, 3 months	PASI75	3 months	11	Repression of inflammatory signals without alteration of the antimicrobial response.
Brodmerkel et al., 2019 [155]	UST	Microarray	Lesional skin	Baseline, 3 months	PASI75	3 months	19	UST ameliorates the perturbed TNF signaling stronger than ETA.
Visvanathan et al., 2019 [156]	RIS, UST	RNA-seq	Lesional skin	3 months	PASI75	3 months	81	RIS induces increased molecular reversion in contrast to UST.
Sofen et al., 2014 [157]	GUS	Microarray	Lesional skin	3 months	PASI75	3 months	24	Repression of T-cell related gene expression.
Lu et al., 2022 [158]	GUS	RNA-seq	Lesional skin	1 month	PASI75	11 months	37	Fast GUS responce is associated to chemotaxis.
Additional approaches								
Zhu et al., 2020 [159]	GUS	Pharmacokinetics	Plasma	1 month	PASI75	1 month	16	GUS does not interact with CYP450 probe substrates.
Loesche et al., 2018 [160]	UST	16s rRNA-seq	Lesional skin	Baseline, 3 months	PASI75	3 months	114	UST displays a body-site-specific microbiome alteration.
Paolino et al., 2022 [161]	UST	EVs	Plasma	n.s.	n.s.	n.s.	10	UST reverses lipid profiles of circulating exosomes.

Abbreviations: n.p., non-pertinent; gDNA, genomic DNA; PASI, psoriasis area severity index; SNP, single nucleotide polymorphism; UST, ustekinumab; RIS, risankizumab; GUS, guselkumab; ETA, etanercept; RNA-seq, RNA sequencing; EVs, extracellular vesicles.

**Table 5:** Pharmaco-omic approaches applied in anti-IL17 agents.

Study, Year	Drug	Method	Sample	Time Sample	Clinical Outcome	Time Point	Sample Size	Main Results
Pharmacogenetics								
Anzengruber et al., 2018 [162]	SEC	Genotypic	n.p.	n.p.	PASI75	3 months	18	No evidence for association with the *HLA-Cw6* allele.
Costanzo et al., 2018 [163]	SEC	Genotypic	n.p.	n.p.	PASI75	6 months	431	No evidence for association with the *HLA-Cw6* allele.
Papini et al., 2019 [164]	SEC	Genotypic	n.p.	n.p.	PASI75	18 months	431	No evidence for association with the *HLA-Cw6* allele.
van Vugt et al., 2020 [165]	SEC, IXE	Genotypic	n.p.	n.p.	PASI75	3 months	134	No evidence for association with *IL-17A* SNPs.
Morelli et al., 2020 [166]	SEC	Genotypic	n.p.	n.p.	PASI75	14 months	62	Association of 8 *HLA-C*, 3 *MICB-DT*, *DDX58* rs34085293 and *TYK2* rs2304255 SNPs.
Pharmacotranscriptomics								
Krueger et al., 2012 [167]	IXE	Microarray	Lesional skin	1 month	PASI75	1 month	46	IXE displays a dosage-dependent efficacious profile.
Wang et al., 2014 [168]	IXE	Microarray	Plasma	2 weeks	PASI75	2 weeks	n.s.	Repression of genes associatied with artherosclerosis after IXE treatment.
Bertelsen et al., 2020 [169]	SEC	Microarray	Lesional skin	Baseline, 2 weeks, 1 month	PASI75	1 month	14	SEC rapidly reverses the NF-κB expression.
Liu et al., 2022 [170]	SEC	RNA-seq	Lesional skin	3 months	PASI75	3 months	15	Incomplete molecular reversion despite the high efficacy levels of SEC.
Seeler et al., 2022 [171]	SEC	ncRNA analysis	Lesional skin	Baseline, 4, 14, 42, 84 days	PASI75	2 months	14	Insights to the regulome of SEC therapy.
Russel et al., 2014 [172]	BRO	Microarray	Lesional skin	Baseline, 2 weeks, 1 month	PASI75	1 month	25	BRO displays a complete molecular reversion.
Tomalin et al., 2019 [173]	BRO	Microarray	Lesional skin	Baseline, 3 months	PASI75	3 months	116	BRO treatment reports a rapid molecular reversion of keratinocyte proliferative marker and higher efficacy versus UST.
Additional approaches								
Piros et al., 2021 [174]	SEC, IXE	Metabolomic profiling	Plasma	Baseline, 6 months	PASI75	6 months	35	Amelioration of circulating inflammatory biomarkers, including C-reactive protein and cholesterol levels after anti-IL17 therapy.
Cao et al., 2021 [175]	IXE	Metabolomic profiling	Plasma	Baseline, 3 months	PASI75	3 months	117	IXE treatment restores the perturbed metabolomic profile, significantly reducing the risk for cardiovascular events.
Zheng et al., 2022 [176]	SEC, ADA	Blood biochemistry	Plasma	Baseline, 4 months, 12 months	PASI75	12 months	196	SEC reduces serum uric acid levels in contrast to ADA.
Yeh et al., 2019 [177]	SEC, UST	16S rRNA-seq	Stool	Baseline, 3 months, 6 months	PASI75	6 months	34	SEC leads to improvement of the microbial profile.

Abbreviations: n.p., non-pertinent; gDNA, genomic DNA; PASI, psoriasis srea severity index; SNP, single nucleotide polymorphism; SEC, secukinumam; IXE, ixekizumab; BRO, brodalumab; ADA, adalimumab; UST, ustekinumab; RNA-seq, RNA sequencing.

## Data Availability

Not applicable.
